# Food Pattern, Lifestyle and Diabetes Mellitus

**DOI:** 10.5812/ijhrba.8725

**Published:** 2014-03-10

**Authors:** Sara Rahati, Mansour Shahraki, Golnaz Arjomand, Touran Shahraki

**Affiliations:** 1 Department of Nutrition, Nutrition Faculty, Tehran University of Medical Sciences, Tehran, IR Iran; 2 Department of Nutrition, Zahedan University of Medical Sciences, Zahedan, IR Iran; 3 Department of Pediatrics, Faculty of Medicine, Zahedan University of Medical Sciences, Zahedan, IR Iran; 4 Children and Adolescent Health Research Center, Zahedan University of Medical Sciences, Zahedan, IR Iran

**Keywords:** Food Handling, Diabetes Mellitus, Type 2, Life Style, Epidemiologic Studies, Clinical Trial

## Abstract

**Background:**

Prevalence of Type 2 diabetes is increasing rapidly worldwide. Recent data is reprehensive of increasing diabetes prevalence from 285 millions in 2010 (6.4%) to 439 millions in 2030 in adults aged 20 to 79 in different countries. Lifestyle and particularly dietary habits play an important role in the development of diabetes. Additionally, specific individual food groups and diet components such as monounsaturated fatty acids, fruits, vegetables, whole grain cereals, dietary fiber, fish, magnesium and nuts may protect against the development of diabetes, possibly through the amelioration of insulin sensitivity and its anti-inflammatory actions, while consumption of red and processed meats and saturated fat may increase the risk of type 2 diabetes.

**Objectives:**

In this section, we studied dietary and other factors related to the effect of lifestyle in type 2 diabetes. These factors may affect the incidence of type 2 diabetes which could be corrected by lifestyle modifications.

**Results:**

Unfortunately, dietary habits in the developed and developing countries are changing towards an unhealthier direction. Consequently, emphasis should be given on encouraging at population and individual levels for adopting a healthier lifestyle, including dietary habits, to prevent the development of type 2 diabetes. Here we reviewed epidemiologic and clinical trial evidence regarding nutrients, foods and dietary patterns to diabetes risk and involved possible mechanisms.

**Conclusions:**

Type 2 diabetes is increasingly growing in young population of developing countries, which causes a large burden on individuals and the society.

## 1. Background

Diabetes is a difficult disease to treat and manage throughout the world (1). Its effects can seriously reduce the life expectancy to 10 years (1, 2). Recent data is reprehensive of increasing diabetes prevalence from 285 millions in 2010 (6.4%) to 439 millions in 2030 in adults aged 20 to 79 in different countries.

Type 2 diabetes is the fourth or fifth major cause of death in most developed countries, and there is growing evidence that it has reached epidemic proportions in many developing countries (3, 4). Type 2 diabetes results from an interaction between a genetic aptitude, high-risk behaviors and environmental risk factors (5). Several lifestyle factors affect the incidence of type 2 diabetes. Obesity and weight gain significantly increase the risk (6-8), and physical inactivity further elevates the risk regardless of obesity (9, 10). Cigarette smoking is associated with a small increase (6, 11), and moderate alcohol consumption with a decrease (11, 12) in the risk of diabetes. In addition, a low fiber diet with a high glycemic index has been associated with an increased risk of diabetes (13), and specific dietary fatty acids may differentially affect insulin resistance and the risk of diabetes (14, 15). It is also known that lifestyle, and particularly dietary habits, play an important role in the development of diabetes (1).

## 2. Objectives

The purpose of our systematic review was to examine epidemiologic and clinical trial evidence regarding nutrients, foods and dietary patterns to diabetes risk and possible involved mechanisms.

## 3. Results

In this investigation, we studied dietary and other factors related to the effect of lifestyle on type 2 diabetes. These factors may affect the incidence of type 2 diabetes, which could be corrected by lifestyle modifications.

### 3.1. Lifestyle Factors

#### 3.1.1. Obesity

Obesity is a powerful predictor for development of type 2 diabetes (3, 16). Obesity has increased in many countries in recent years (17). Diabetes is resulted from an interaction between environmental and genetic factors. These factors include: physical inactivity (3), habitual energy intake in relation to expenditure (3); and macronutrient composition of the diet (18, 19) and metabolic characteristics (3). Increase in obesity is associated with increased incidence of type 2 diabetes (20).

In a study reported. Although weight gain during adolescence leads to adulthood obesity (21). Increase in overweight and obesity in adulthood is associated with increased risk of type 2 diabetes. In a study conducted by Shahraki and colleagues low education level was found as a strong determinant of overweight and obesity among Iranian women (22).

Nurses healthy study suggested a lower risk of diabetes in subjects with BMI below 21 (7). Only a limited number of studies had specific criteria for age and sex, as the prevalence of diabetes has been associated with obesity. Those with higher BMI have higher incidence rates of type 2 diabetes at earlier ages than those with lower BMI among whom the incidence rises in older age groups (16).

Some studies suggested that waist circumference or waist-to-hip ratio is a better predictor for the incidence of diabetes (23) and cardiovascular risk factors at different age groups than BMI (24). Some data suggested that distribution of body fat is an important risk factor indicative of abdominal obesity or visceral fat. For example, In Japanese American men intra-abdominal fat, measured by CAT scan, was the best anthropometric predictor of diabetes incidence (25). Given the role of central adiposity as a determinant for the risk of diabetes, it is necessary to know the normal BMI (18.5–24.9 kg/m^2^) for all populations. Some studies have shown that the risk of diabetes increases with the normal BMI of 21 kg/m^2^ (7).

Pacific people have a greater proportion of lean body mass than Europeans; therefore, a higher BMI cut off may be acceptable for this population. Because data on waist circumference and waist-to-hip ratio are not consistent, so it is best to use the WHO recommended BMI range (18.5-24.9 kg/m^2^) and population mean of 21 kg/m^2^ (26).

#### 3.1.2. Physical Inactivity

Exercise as a series of planned and repeated actions of skeletal muscles is associated with energy consumption (27). Numerous studies have shown an association between physical inactivity and the incidence of type 2 diabetes (3, 27). Exercise has a significant role in the regulation of blood glucose, metabolism of proteins and fats, improvement of insulin action, prevention of complications of diabetes, improvement of muscle flexibility and strength, beneficial effects on the cardiovascular system and increasing life expectancy of patients. In addition, physical activity is beneficial for the mental state of individual, because it increases the energy of the human body, improves self-esteem and decreases depression (28).

The basis of a useful exercise is its intensity, duration and frequency. The duration of the exercise should be 30 minutes in the beginning, starting with 5-10 minutes of warm-up and ending always with recovery exercises. The lower frequency recommended is 3 times/wk. Usually, low-intensity and long-duration exercise programs are considered as the most appropriate and safe patterns for patients with diabetes (27).

### 3.2. Dietary Factors

#### 3.2.1. Carbohydrates

The optimal and normal carbohydrate to lipid ratio in diet is a major challenge considering its role to prevent chronic diseases such as type 2 diabetes (29). In a study conducted by Richard et al. it was observed that reduced dietary fat intake and increased intake of carbohydrates prevent the incidence of chronic diseases (30). Some studies demonstrated that increased intake of carbohydrates reduced the incidence of diabetes (31, 32). However, several studies reported that increased carbohydrate intake would decrease HDL levels and increase fasting plasma TG concentrations (33). Recently, two cohort studies (34, 35) and one review study (36) found no association between total received carbohydrate and the risk of diabetes. These data suggested that increased carbohydrate intake increases the secretion of insulin to maintain insulin homeostasis, and a high carbohydrate intake, leading to insulin secretion, is associated with receiving energy that causes higher levels of insulin after a meal. Insulin secretion with high output may be associated with age-related decline in insulin secretion, resulting in a more rapid development of diabetes (37).

Findings from epidemiological and metabolic studies regarding the association of dietary carbohydrates and fiber with diabetes are inconsistent (38). Several metabolic studies supported the useful (39), neutral (40, 41) and harmful (42) effects of carbohydrate-rich diet compared to a high-fat diet on glycemic response. The results of ecological and cross-sectional studies showed that high-carbohydrate diet reduces the incidence of type 2 diabetes (31, 43), while the results of cohort studies did not support any association between diabetes and total dietary carbohydrate (44).

It was reported that dietary fiber, particularly soluble fiber, improves the postprandial glycemic response and insulin concentration through slowing down the digestion and absorption of food and creating a gel-like substance in the stomach by several metabolic hormones (38). Several studies have shown that glycemic control is improved and LDL cholesterol decreases with relatively high carbohydrate, low fat diets including naturally occurring fiber-rich foods compared with relatively low carbohydrate, higher fat diets (45). Clinical studies on glycemic index and glycemic load also showed that the form and content of carbohydrate and fat intake may be effective in short-term glycemic response (38). In two cohort studies, a direct association was found between glycemic index and glycemic load with the risk of diabetes (3). Most recent American dietary guidelines recommend consumption of a variety of grain products, especially whole grains at least six servings a day or more. WHO/FAO recommended to get at least 55% of energy intake form carbohydrate in normal people (46, 47). Hence, there are no specific carbohydrate guidelines to prevent diabetes. Thus, receiving an extensive range of carbohydrates may reduce the risk of type 2 diabetes, depending on the type and source of received carbohydrate compared to its amount (46).

#### 3.2.2. Fat

Quantity and quality of dietary fat affect glucose tolerance and insulin sensitivity (3, 48). A high fat diet may cause glucose intolerance through several mechanisms, including lowering insulin binding to its receptors, degradation of glucose transport, reducing TG synthesis, and accumulation of stored triglycerides in skeletal muscles (49, 50). The fatty acids composition may be related to insulin function through its effect on composition of membrane’s phospholipids, which in turn affect membrane fluidity and insulin signaling (51).

#### 3.2.3. Amounts of Fat Intake

Animal studies showed that consumption of high fat diet except for n-3 fatty acids is associated with insulin resistance compared to the high-carbohydrate diet (52). In two cross-sectional studies, total fat intake in individuals with glucose intolerance and type 2 diabetes as well as those with gestational diabetes were higher compared to individuals with controlled glycemic index. Therefore, a high-fat diet is a good predictor of developing IGT in healthy people as well as IGT development to type 2 diabetes (31, 53). High intake of total fat is associated with increased fasting insulin concentration and decreased insulin sensitivity index (54). On the other hand, several studies showed no association between total fat intake and the risk of diabetes (55, 56).

#### 3.2.4. Nature of Dietary Fat

As noted above, in animal studies, intake of saturated fatty acids, monounsaturated and polyunsaturated fatty acids except for the n-3 fatty acids, led to insulin resistance when consuming a high-fat diet (3). Epidemiological studies suggested that high intake of saturated fat is associated with the risk of IGT and increased fasting glucose and insulin levels (57). The higher proportion of saturated fatty acids in serum lipids or phospholipids in muscles associated with higher fasting insulin levels would reduce insulin sensitivity (58, 59) and increase the risk of type 2 diabetes (59). Higher intake of vegetable fat and PUFA reduces the risk of type 2 diabetes as well as decreased fasting plasma glucose concentration and the two-hour glucose concentration (56). Therefore, a higher proportion of long-chain polyunsaturated fatty acids in phospholipids of muscles would improve the insulinemic sensitivity in humans (49). Nevertheless, human data are inconsistent regarding mono-unsaturated fatty acids. Some studies suggested that receiving more mono-unsaturated fatty acids would increase the risk of type 2 diabetes. However, monounsaturated fatty acids are not resulted from vegetable oils in the western diet, but they have a grand symbiosis with saturated fatty acids in meat and dairy sources that the harmful effects might be due to the presence of saturated fats (60).

In a short-term human study, replacing a substantial portion of saturated fatty acids with unsaturated fats improved glucose tolerance in healthy young and middle-aged women with hyperglycemia, while replacing saturated fatty acids with mono unsaturated fatty acids had no positive impact on those receiving fat for more than 37% of their daily energy (61).

### 3.3. Micronutrients

#### 3.3.1. Vitamin E

A case-control study associated with a cohort study showed that people with high blood levels of vitamin E are 39% at lower risk of diabetes than those with lower serum levels of vitamin E. It was reported that reduced plasma levels of antioxidant vitamins are associated with increased risk of chronic diseases (3, 62).

#### 3.3.2. Magnesium

Magnesium is a component of grains found in the shell of cereals (38). Three large cohort American studies reported a strong negative correlation between magnesium intake and the risk of type 2 diabetes (34, 35, 56), while another cohort study on 45-64 years old men and women showed no association between magnesium intake and the risk of type 2 diabetes. However, because the latter was smaller than the other three studies in scale, it would be statistically weaker (63).

#### 3.3.3. Chromium

Vincent et al. reported that consumption of chromium supplementation in people with mild glucose intolerance improved glucose tolerance and decreased the blood levels of insulin. However, it was not seen in people with normal glucose tolerance (64). Reduced blood insulin levels suggest improved tissue sensitivity to insulin due to chromium supplementation (3).

### 3.4. Mediterranean Diet and Diabetes

Mediterranean diet was introduced for the first time in 1960s by Ancel Keys through observing food habits of Mediterranean populations (65). The Mediterranean dietary pattern emphasizes a consumption of fat primarily from foods high in monounsaturated fatty acids and mainly olive oil and encourages daily consumption of fruits, vegetables, low fat dairy products and whole grains, weekly consumption of fish, poultry, tree nuts, legumes, monthly consumption of red meat, as well as a moderate consumption of alcohol, high ingestion of dietary fiber, antioxidants, polyphenols and magnesium (65, 66). In addition, it normally contains meals, but the proportions of macronutrients may vary. There is no single Mediterranean diet, although the dietary patterns in the Mediterranean region have many common characteristics.

The Mediterranean diet is one of the best-known food patterns for the human health. The Mediterranean diet has beneficial effects for the prevention of type 2 diabetes. These effects include reduced oxidative stress and insulin resistance. Mediterranean diet can act as an anti-inflammatory dietary pattern able to maintain or treat chronic diseases, such as type 2 diabetes (66). 

## 4. Conclusions

Type 2 diabetes is increasingly growing in young population of developing countries, which causes a large burden on individuals and the society. Therefore, prevention of diabetes should be considered as a priority as follows:

Development and evaluation of healthy lifestyle plans, focusing on the following aspects: 

Prevention and early treatment of overweight and obesity, especially in high risk groups.

Consuming a nutritious diet including low-fat content, especially saturated fat, no sugar and high NSPs.

Active lifestyle including regular physical activity at least an hour a day, and vigorous activities necessary to reduce the risk of type 2 diabetes.

Moderate intake of alcohol and quit smoking.

Rapid identification of individuals at risk of type 2 diabetes.

Identifying individuals at risk of high blood pressure, diabetes and heart disease.

Screening for gestational diabetes.

Maternal nutrition and maintaining weight.

Healthy lifestyle programs and their interventions should be specified for each age group and their developmental stages.
